# Evaluation of Waste Related to the Admission Process of Low-Complexity Patients in Emergency Services, in Light of the Lean Healthcare Philosophy

**DOI:** 10.3390/ijerph19127044

**Published:** 2022-06-08

**Authors:** Letícia Bianchini de Barros, Laura Passos Caldas, Elena Bohomol, Alice Sarantopoulos, Vinicius Minatogawa, Renata Cristina Gasparino

**Affiliations:** 1 School of Nursing, University of Campinas, Campinas 13083-887, Brazil; grenata@unicamp.br; 2 School of Nursing, Pontíficia Universidade Católica de Campinas, Campinas 13087-571, Brazil; la.passos@outlook.com; 3 School of Nursing, Federal University of São Paulo, São Paulo 04024-002, Brazil; ebohomol@unifesp.br; 4 Atos Inova Saúde, Campinas 13085-055, Brazil; alice_sarantopoulos@hotmail.com; 5 Escuela de Ingeniería de Construcción y Transporte, Pontificia Universidad Católica de Valparaíso, Valparaíso 2340000, Chile; vinicius.minatogawa@pucv.cl

**Keywords:** process assessment, health care, quality management, health services administration, emergency service, hospital, hospitals, university

## Abstract

The adequacy of work processes in healthcare services contributes to the quality of care provided to the patient. However, in emergency units, overcrowding is a constant reality, resulting in the lack of materials and long waiting lines. Taking this into consideration, this study aimed to map and analyze the value stream of patients classified as blue, green, or yellow in a Referral Emergency Unit. The evaluation research with analysis of processes was carried out in a teaching hospital on 30 patients seen at the emergency service. Value Stream Maps were drawn and the times involved in the process were calculated. Wastes and their possible causes were identified. A total of 13 maps were created and the mean process time between the activities involved in the process ranged between 7.3′ and 114.0′; the interruption time, between 0′ and 27.6′; the waiting time, between 43.2′ and 507.5′; and the lead time between 56.6′ and 638.0′ min. Some causes of waste were: high demand from patients; a shortage of personnel and offices. Following the Ishikawa Diagram, most of the waste is found regarding methods, human resources, and physical structure.

## 1. Introduction

Emergency units are intended to provide early care and adequate transport of patients, organizing the care flow [1]. However, due to overcrowding, structural, and administrative difficulties, the organization of this system is poor [2]. For emergency units to efficiently and quickly meet their demands, they must have an organized administration, with good quality materials, in sufficient quantity and a staff of trained and qualified professionals to provide care [3].

Overcrowding of emergency services is one of the main triggers of adversity in these units, as it leads to disorganization of work and, consequently, impairs the quality of care and hospitalizations [4]. Seeking to improve the flow of patients within the service, Patient’s Risk Classification Protocols were created, which establish patient care according to the severity of their clinical condition or complaint; among them, the Manchester Triage System stands out [5].

This protocol consists of a system composed of five levels of care classification represented by different colors (red, orange, yellow, green, and blue). Each color indicates an estimated care time and priority. Patients classified as red have the highest priority and must be attended to immediately and the patient classified as blue can wait up to 240 min, being the one with the lowest priority. To delimit this priority, systematic severity criteria are analyzed, such as patient complaints, clinical history, signs and symptoms, vital signs, and physical conditions. Once analyzed, it is possible to prioritize the case and estimate a waiting time target for assistance [6].

According to the literature, most emergency overcrowding results from the spontaneous search for the service by low-complexity patients, generally classified as blue, green, and yellow [7]. Considering that patients classified according to these colors do not require health devices of high technology, they should be seen in services with low technological complexity [8]. Hence, it is evidenced that classification protocols assist in the service organization. Nevertheless, they do not reduce overcrowding. To do so, it is necessary to evaluate work processes that can be implemented according to the Lean philosophy [9].

Lean Thinking was developed based on the Toyota Production System, and its main focus is the elimination of unproductive and repetitive activities by implementing standardized and sustainable work processes. Its principles consist of determining the meaning of value for customers, identifying the value stream, maintaining this continuous flow, a pull system, and implementing a culture of continuous improvement (Kaizen) [10].

These principles have been adopted by the healthcare area, and the Lean Healthcare method aims at improving the patients’ health status and their satisfaction with the received care. To do so, there must be an adequate administration of inputs, time, and human resources, seeking to gradually eliminate wastes and improve processes that add value to the patient [10,11]

The concept of “value” is defined by patients themselves, and the processes must meet their needs and be in accordance with their preferences and adapted to their demands. To add value, a continuous workflow is necessary, which enables one to visualize activities that have value, which ones are necessary, though without adding value, and the wastes of the process [12].

One of the Lean tools that enable evaluating the flow is the Value Stream Mapping (VSM), which assists in diagramming and documenting all steps of the processes throughout hospital departments, that is, in the case of the patients, they will be monitored from their admission to their departure from the healthcare service, in such a way it is possible to analyze what adds value and the waste within the flow [13].

To develop a VSM, it is necessary to use specific metrics, such as the lead time. The lead time aims to analyze the time window from the patient’s arrival to the unit until its departure. That is, this is the duration of the whole process. It is entirely related to other metrics considered by the VSM approach. For instance, the wait time and downtime [14]. In the methodology section, we present the metrics included in this study.

This analysis will allow identifying waste and its causes, as well as its impacts. This will have a positive and direct impact on processes that add value to the patient. When wastes are eliminated, reinvestments in terms of time and finance can be made in other areas of the healthcare service [15].

The identification of possible wastes in the risk classification process of patients who need low-complex technologies for their care can contribute to the organization of processes, reduction of overcrowding and long waiting lines, and better use of the physical structure and materials, bringing benefits not only to patients but also to professionals and different institutions [16].

Hence, this study seeks to understand what wastes are verified in the flow of care for patients who need low-complex technologies in a referral emergency unit. To do so, the objectives consist of mapping and analyzing the value stream of patients classified as blue, green, or yellow in a Referral Emergency Unit.

## 2. Materials and Methods

This study uses as a methodological framework the Evaluation Research, with an analysis of processes [17], aiming to obtain information on the current status of the flow of patients classified as blue, green, or yellow in a Referral Emergency Unit (REU).

This REU belongs to a large Brazilian public hospital, a reference for urgent and emergency care in its city and region. According to the institutional indicators of 2019 (pre-pandemic period), the unit served, on average, 156 patients/day, with 74.7% seeking the service by spontaneous demand; the mean length of stay was 15 h, and the occupancy rate was 157.4%.

The study was conducted on 30 patients who were monitored from admission to hospitalization or departure; they were selected by convenience, aged over 18 years, and were classified as blue, green, or yellow, according to the Manchester Triage System, in the period between November of 2019 and January 2020. Exclusion criteria were: patients classified as blue, green, or yellow but who were reclassified as orange or red during the care process.

Patients were approached by the researchers after having their risk classified by the nurse of the unit. When classified as blue, green, or yellow, researchers introduced themselves, explained the research objectives, and those who agreed to participate in the study signed an Informed Consent Form.

As of that moment, the researchers monitored and observed all the steps taken by the patient, from their admission to the unit to their departure or hospitalization. A characterization form was applied, which addressed data such as age, sex, classification, specialty, and patient outcome. During the observation of the patient’s journey, the following times were timed according to the Lean methodology [11,12]:

- Process Time (PT): time required to complete a process step;
- Waiting Time (WT): time waited by the patient between steps of the process;
- Interruption Time (IT): time in which care is interrupted within each activity performed in a process;
- Lead Time or Duration Time (LT): total time of the process;
- Percentage of value-added (VA): reflection of the value-added generated by the process to the patient.

After data collection, the VSM were drawn, wastes were identified, and their possible causes were analyzed according to the Ishikawa Diagram, together with the team of professionals from the REU. Data related to times were tabulated in Excel for the Windows^®^ program. Absolute and relative frequencies of categorical variables and measures of position (minimum, maximum, mean, and median) and dispersion (standard deviation) of continuous variables were calculated, in addition to the design of the VSM according to the collected data. To calculate the LT, the PT, WT, and IT of all activities involved in the process were added. The percentage of value-added was obtained by dividing PT by LT (PT ÷ LT).

## 3. Results

Thirty patients participated in the study, 15 (50%) women and 15 (50%) men. According to the Manchester Triage System, 13 (43.3%) patients were classified as yellow; 8 (26.6%) as green, and 9 (30.0%), like blue, with 26 (86.6%) patients from the Internal Medicine medical specialty; 2 (6.6%) from Surgery; 1 (3.3%) from Orthopedics; and 1 (3.3%) from Neurology. The mean age of patients was 55.8 (±15.2) years.

Considering patients classified as yellow, some examples of complaints were: chest pain, abdominal pain, hematemesis, and poor general condition in the elderly. For patients classified as green, complaints such as epigastric pain, headache, fever, and fecal incontinence appeared. Patients classified as blue had complaints such as joint pain. Three left the service without being attended, eleven were referred to other services, four were hospitalized and twelve were discharged.

All patients spontaneously sought the service and were initially welcomed by a security guard or a nursing technician. These professionals instructed patients to wait for registration in the system, which was carried out by an administrative assistant. Subsequently, they underwent the Risk Classification, carried out by a nurse following the Manchester Triage System, in a separate area located a few meters from the main entrance to the REU. In case patients were classified as blue or green, they would wait for medical screening; however, if they were classified as yellow, the nurse would immediately refer the patients to care within the unit.

**Figure 1:**
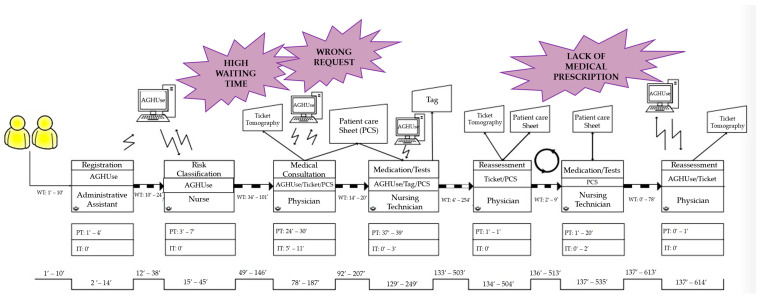

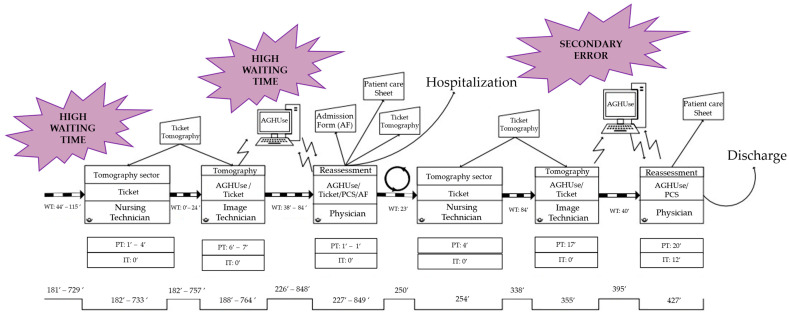
Value Stream Map of patients who required tomography (*n* = 2).

**Figure 2:**
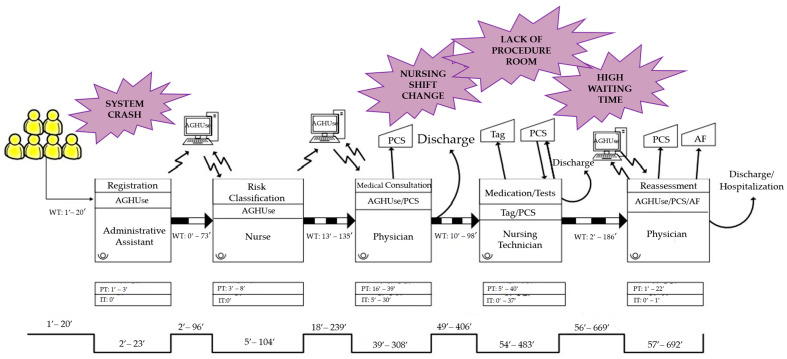
Value Stream Map of patients who required medication (*n* = 6).

Different flows were followed as of the moment the blue, green, or yellow patient entered the room; therefore, 13 VSM were drawn, and, later, the wastes were identified. These maps, with their respective wastes, were distributed into five groups to facilitate the illustration of the process: (1) one map for patients who left the service without being seen; (2) two maps for patients referred to other services after medical screening; (3) one map for patients who required tomography (Figure 1); (4) two maps for patients who required medication (Figure 2), and (5) seven maps for patients who underwent X-ray and electrocardiogram (ECG).

In the first group, patients who left the service without being seen, a map was drawn for three patients; two of them were classified as green and one as blue. These patients had been classified by the nurse and were waiting for the medical screening; however, the three patients left before the medical consultation. The times measured during this process are presented in Table 1.

The map concerning the third group, composed of two patients who required tomography, is represented in Figure 1. In the fourth group, patients who required medication, two maps were drawn. The first represented the flow of a patient classified as green, whose flow differed from the others, and the other map (Figure 2) contains the flow of six patients, all classified as yellow. The mean process, interruption, waiting, and lead times and value-added concerning each of the five groups of maps are represented in Table 2.

The mean times regarding all activities included in the mapped process, considering the total number of patients who were involved in each one of them, are represented in Table 3.

**Figure 3:**
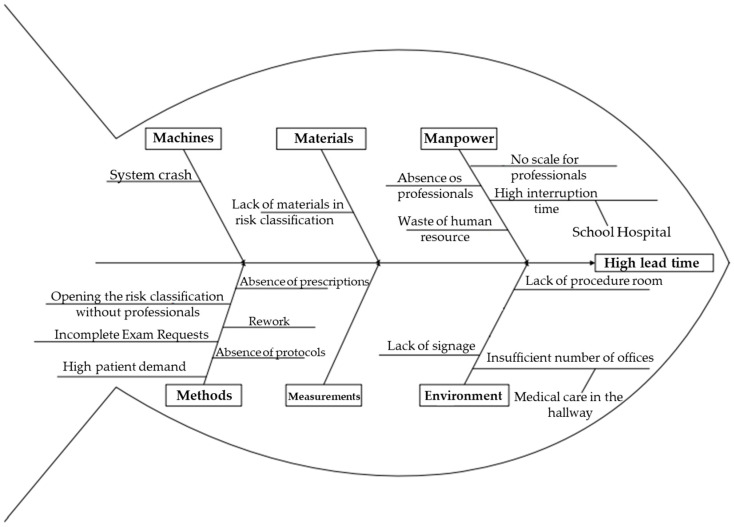
Analysis of possible causes of wastes verified in Value Stream Maps.

After the preparation of maps and tables, the possible causes of the verified wastes were analyzed using the Ishikawa Diagram. The main results are shown in Figure 3.

## 4. Discussion

The following discussion will jointly address the groups of maps drawn with their respective wastes and possible causes. The causes of waste were analyzed together with the healthcare professionals of the unit.

The need for multiple maps to represent a single process—from admission to departure/hospitalization of low-complexity patients—may reflect the study sample size; however, authors report that although the VSM is widely used in Lean Healthcare applications, there is no formal recommendation on how to make this calculation [18]. Taking this into consideration, the authors of the present study chose to use a larger sample than that of other studies [19,20], aiming to understand the process better.

Moreover, it is noteworthy that this high number of maps may also reflect a lack of standardization, as even in cases in which patients had similar clinical complaints and needs, they followed different flows within the process, demonstrating a lack of well-established protocols.

Regarding groups of the maps, in group 1 (patients who left the service without being seen), the greatest waste verified was related to longer WT, which contributed to a considerable LT and, consequently, to low value-added. No articles whose authors drew VSM for patients in this situation were found in the literature. Apparently, these studies disregarded such patients, possibly due to differences in the research objective. However, the authors of the present study consider it important to present them, as departure rates reflect the overcrowding of the service due to longer WT [21], which may explain the findings of this study, considering that all patients who left had waited between one and five hours and received no medical care.

When analyzing the possible causes for this waste, with the aid of the Ishikawa Diagram, problems regarding methods and manpower were detected, considering that the nurse was providing care before the arrival of the physician. As doctors are responsible for making the decision to discharge or refer these patients to other services [22], multidisciplinary work is of paramount importance. The absence of physicians at the beginning of the shift was attributed to the lack of a work schedule for these professionals, in addition to the understaffing of the service. According to the literature, the poor distribution and insufficiency of personnel are sources of waste and entail great costs for institutions, in addition to compromising patient safety [23].

Regarding group 2, patients who were referred to other healthcare services all of them were classified as green or blue. When analyzing the causes, as for the method, the high demand from patients who did not require complex technologies for their care was listed as the cause of these referrals. In addition, the long WT in these maps is due to the lack of materials used for risk classification, as nurses often had to look for these materials in the unit.

Studies that address the low problem-solving capacity of the Health Care Network state that patients should initially seek services with low technological complexity, that is, in primary health care services. However, there is great difficulty in accessing these services; in such a way, patients seek care for their complaints in hospital units, thus overloading them [8]. Conversely, primary health managers report that the precariousness of working conditions, lack of integrative policies, political interference, lack of flow, and clear communication between the many levels of the healthcare system contribute to compromising principles such as comprehensiveness and resoluteness of actions [24].

In group 3, patients who underwent tomography, a similar flow during the first ten steps of the process was verified. However, after these steps, one of the patients had to undergo the examination again due to a mistake made when requesting the test, thus generating rework. Furthermore, the prescription of medication without a controlled substance prescription form was observed on the same map. Researchers also found results similar to those of the present study, with the description of waste including imprecision in request forms for tomography exams, lack of communication between the healthcare teams, and high patient demand [25].

Furthermore, these wastes can be related to the fact that the research was conducted in a teaching hospital, and medical students consider the undergraduate teaching in urgency and emergency to be slightly inadequate [26].

In addition, it was possible to observe a long WT for medical reassessment (1-h), resulting in an LT of approximately 11-h. The cause of this considerable WT was attributed to a structural problem due to the insufficient number of offices. Taking this into consideration and seeking to streamline the provision of care, patients were reassessed in the hallways of the unit, compromising secrecy and privacy of care and, consequently, it’s quality.

In the fourth group, patients who required medication, some wastes that delayed the process were observed, such as shift changes, which happened in a disorganized way. Studies describe that the reduced number of personnel and the work overload, especially in the emergency sector, separate the employee from quality care [27,28]. This fact corroborates the aforementioned waste, as there was only one nursing professional in the medication room. Thus, both patients who were already waiting for assistance in this room and those who had just been referred to take medication had to wait for care, which contributed to an increase in WT and, consequently, in LT.

In this group of patients who required medication, a waste of human resources was surveyed. To receive patients in the unit, there was a nursing technician only assigned to guide the patient to wait for registration and risk classification, a situation that describes a waste of human talent, which consists in not taking advantage of the professional’s technical-scientific knowledge [29].

Moreover, in this VSM, another verified waste was caused by problems with the computerized system. System crash contributed to brief IT, considering that, usually, the system started working again quickly. Nevertheless, on one of the days of data collection, the system was down for hours, and professionals who were already used to using this technology had difficulties handling the problem and providing care without the tool, demonstrating that they do not have contingency measures for the service in a standardized way.

The lack of a specific room for performing procedures was also a cause related to the environment, evidenced-based in the creation of this map, which contributed further to prolonging the patients’ journey in the unit, as they often had to wait with discomfort for an empty room to receive care.

In the last group, patients who underwent X-rays and ECG, each of the seven patients in this group followed a flow with similar steps, but in different orders, in such a way that a VSM was drawn for each one of the patients. The Lean philosophy addresses the need to standardize work processes to achieve continuous improvement by identifying wastes, analyzing their causes, and implementing interventions [10].

The facts verified by drawing these maps, once again, demonstrated the lack of standardization in the clinical management and investigation of cases, which could be solved based on well-established protocols that contribute to the reduction of LT [30]. In addition, it was also possible to verify that the lack of signalization in the unit contributed to the increase in LT, as patients were unable to move within the institution easily.

Based on the creation of the maps, it was possible to table the means of PT, IT, and WT of each activity in which the patient was involved during their journey in the UER. Accordingly, it was observed that the mean WT is higher than the PT in all activities, except for the time undertaking tests and taking medication. This indicates that the amount of time devoted to what actually adds value to the patient (PT) is much smaller when compared with WT, which does not add value.

The cause of the long IT in medical consultations, a datum found in most groups of the maps, was attributed to the fact that this is a university hospital. This time is used for case discussions between students and professors. It is worth noting that despite the IT of the medical consultation being the highest among all activities; the PT was also one of the highest because students take longer to provide care.

According to the literature, these exchanges of information are of great importance for student learning [19]. However, most of the time, there were four students seeing patients and only one professor available to conduct the discussion. This fact contributed not only to increasing the IT but also the WT of other patients who were waiting for their consultation. It is worth highlighting that services with shorter interruption times and leaner processes contribute to a higher quality of care and increased VA for patients [31,32].

Collecting data for drawing VSM is a particularly challenging task, as the researchers could not interfere with the patient flow. It was possible to notice, several times, that they were longing for further clarification. It is also worth mentioning that, as the patients were monitored from their arrival to hospitalization or departure from the institution, the period alongside the patient was very long and often distressing, as the researchers shared the patients’ suffering and fatigue during their stay in the unit. Perhaps these are the reasons why many institutions make use of external consultants aiming not to influence the data that will be collected [19].

## 5. Conclusions

Following the study’s objectives, we developed the Value Stream Mapping of patients classified as blue, green, or yellow in a referenced emergency unit following the study’s objectives. With the support of the VSM tool, we were able to identify waste, mainly related to a long lead time, excessive waiting time, and several interruptions detected during the patient’s journey within the unit. Through the Ishikawa Diagram, it was possible to analyze the causes of the waste found. We noticed that most regard methods, human resources, and physical structure. Combining both tools highlights waste in emergency units in a very clear and visual way.

The work presents relevant contributions to the literature and practices. From a theoretical point of view, the results provide a clear example of how to apply Lean Healthcare tools to diagnose and identify waste in emergency units. Thus, we provide a guide for future applications of Lean Healthcare in other emergency units.

In addition, the present research evaluated patient samples that are not normally found in the literature. Specifically, this study evaluated patients who left the service before receiving medical care. Assessing the journey of such patients can provide data on why they evade before receiving care. As managerial implications, the waste identified in this study and its possible causes will help managers plan and implement changes in behavior, processes, and physical structure. That can result in improvements not only for patients but also for professionals.

It is important to highlight as limitations of the work the focus on diagnosing waste. That is, proposals to reduce the impacts of the bottlenecks found are not addressed. In addition, the work was limited to the analysis of a sample of 30 patients. However, it is important to note that this sample volume is larger than what is seen in the literature on the application of lean healthcare. It is noteworthy that it is not clear an ideal sample size in the literature.

As a proposal for future studies, researchers can apply other Lean tools. Such a proposition would add knowledge of using different approaches to emergency processes. Besides waste identification tools, some methods can focus on reducing waste in low-complexity patients’ care. Another proposal for future studies would be evaluating the satisfaction of patients treated by emergency unities. That would facilitate the process of adding value to the patients, which should be the institution’s primary focus. It is also essential to assess how the lean culture is doing within the unities and how it can be improved. Continuing the application of lean through future studies is key to encouraging continuous improvement.

## Figures and Tables

**Table 1:** Process, interruption, waiting, and lead times and value-added obtained by the Value Stream Maps of patients who left the service without being seen (*n* = 3).

Times (Minutes)	Mean (*n* = 3)	Standard Deviation (*n* = 3)	Minimum (*n* = 3)	Median (*n* = 3)	Maximum (*n* = 3)
Process time	7.3	0.9	6.0	8.0	8
Waiting time	160.7	107.6	65.0	106.0	311
Interruption time	0.0	0.0	0.0	0.0	0
Lead time	168.0	106.7	73.0	114.0	317
Value-added	7%	4%	2%	7%	11%

**Table 2:** Process, interruption, waiting, and lead times and value-added were obtained by the five groups of Value Stream Maps (*n* = 30).

		Process Time				Interruption Time				Waiting Time				Lead Time				VA ^4^
	*n*	Mean	SD ^1^	Min. ^2^	Max. ^3^	Mean	SD ^1^	Min. ^2^	Max. ^3^	Mean	SD ^1^	Min. ^2^	Max. ^3^	Mean	SD ^1^	Min. ^2^	Max.^3^	Mean
**Patients who left the service**	3	7.3	0.9	6.0	8.0	0.0	0.0	0.0	0.0	160.7	107.6	65.0	311.0	168.0	106.7	73.0	317.0	4.3%
**Referred patients**	11	13.3	39.5	6.0	25.0	0.2	0.4	0.0	1.0	43.2	39.5	5.0	140.0	56.6	43.1	14.0	156.0	23.3%
**Patients who underwent tomography**	2	114.0	37.0	77.0	151.0	16.5	11.5	5.0	28.0	507.5	59.5	448.0	567.0	638.0	11.0	627.0	649.0	12.3%
**Patients who received medication**	7	53.7	29.6	10.0	102.0	27.6	22.5	0.0	62.0	259.6	275.4	37.0	912.0	340.9	290.2	47.0	1013.0	15.7%
**Patients who underwent X-ray and ECG**	7	84.4	52.4	28.0	195.0	22.4	17.9	0.0	49.0	192.4	112.1	73.0	431.0	299.2	166.2	123.0	675.0	28.2%

¹ Standard Deviation ² Minimum ³ Maximum ^4^ Percentage of value-added.

**Table 3:** Mean process, interruption, and waiting times of each activity involved in the process (*n* = 30).

		Process Time (Minutes)	Interruption Time (Minutes)	Waiting Time (Minutes)
	*n*	Mean	Mean	Mean
Registration	30	2.8	0.1	5.4
Risk classification	24	4.4	0.0	11.8
Medical screening	11	7.6	0.0	71.3
Classification/Screening	6	4.7	0.0	11.2
Medical consultation	16	28.3	16.8	78.8
Medication/Tests	13	32.5	6.2	28.5
Electrocardiogram	4	5.0	0.0	16.8
X-ray	5	5.6	0.2	34.6
Tomography sector	2	4.5	0.0	91.0
Tomography	2	15.0	0.0	54.0
Medical reassessment	14	8.6	2.4	119.8
